# Peer selection and influence effects on adolescent alcohol use: a stochastic actor-based model

**DOI:** 10.1186/1471-2431-12-115

**Published:** 2012-08-06

**Authors:** Marlon P Mundt, Liesbeth Mercken, Larissa Zakletskaia

**Affiliations:** 1 Department of Family Medicine, University of Wisconsin School of Medicine and Public Health, 1100 Delaplaine Ct, Madison, WI 53715, USA; 2 Department of Primary Care Health Promotion, Maastricht University (Netherlands) School for Public Health, PO Box 616, 6200, MD, Maastricht, the Netherlands

## Abstract

**Background:**

Early adolescent alcohol use is a major public health challenge. Without clear guidance on the causal pathways between peers and alcohol use, adolescent alcohol interventions may be incomplete. The objective of this study is to disentangle selection and influence effects associated with the dynamic interplay of adolescent friendships and alcohol use.

**Methods:**

The study analyzes data from Add Health, a longitudinal survey of seventh through eleventh grade U.S. students enrolled between 1995 and 1996. A stochastic actor-based model is used to model the co-evolution of alcohol use and friendship connections.

**Results:**

Selection effects play a significant role in the creation of peer clusters with similar alcohol use. Friendship nominations between two students who shared the same alcohol use frequency were 3.60 (95% CI: 2.01-9.62) times more likely than between otherwise identical students with differing alcohol use frequency. The model controlled for alternative pathways to friendship nomination including reciprocity, transitivity, and similarities in age, gender, and race/ethnicity. The simulation model did not support a significant friends’ influence effect on alcohol behavior.

**Conclusions:**

The findings suggest that peer selection plays a major role in alcohol use behavior among adolescent friends. Our simulation results would lend themselves to adolescent alcohol abuse interventions that leverage adolescent social network characteristics.

## Background

Early adolescent alcohol use is a major public health challenge. One quarter of all adolescents begin drinking alcohol by 13 years of age [1]. Drinking before the 14th birthday is associated with a fourfold increase in risk of alcohol dependence in adulthood [2]. Early alcohol initiation is linked to many risky adolescent behaviors, including marijuana and cocaine use, having sex with multiple partners, and academic underperformance [3].

A wide body of literature indicates that adolescents and their friends exhibit more similar alcohol use behavior than would be expected by chance alone [4,5]. Drinking by a best friend has been tied to alcohol initiation among middle and high school students [6,7]. In a systematic review of longitudinal studies on adolescent drinking, alcohol-using peers were consistently predictive of an adolescent’s own drinking behavior at a later wave [8]. However, there is debate over the mechanism by which friends come to resemble one another over time. One possible explanation is that similarities occur as a result of peer influence, or the spread of behaviors and behavioral norms through social ties. In this manner, the behavior of an individual would move toward the average behavior of one’s friends over time. Another pathway to homogeneity within friendships is that friends may be similar due to social selection, or homophily, the tendency for similar people to be attracted to and form friendships among one another.

Understanding the mechanism by which similarity in alcohol use behavior among adolescent friends occurs is an important clinical matter. Lacking clear direction on the causal pathways between peer interactions and alcohol use, adolescent brief alcohol interventions have produced, at best, mixed results [9,10]. As an example, influence-driven contagion in adolescent groups would lend itself to peer-to-peer methods of alcohol use intervention. It would also allow for the possibility of multiplier or spillover effects from targeted individuals to a larger network of friends. Selection-driven behavior patterns in adolescent groups would lend themselves to interventions that target alcohol use in adolescent friendship groups identified through social network characteristics.

Previous studies have attempted to disentangle selection from influence effects in adolescent alcohol use [8]. Analytic techniques employed have included structural equation modeling [11-13], latent growth models [14-18], instrumental variables [19], and fixed effects [20]. These studies have relied on lagged indicators of alcohol use and friendship connections in an attempt to isolate the selection effect from influence. The results indicate that both selection and influence are occurring, but the relative contribution of the two factors cannot be determined.

A major limitation in prior studies on selection and influence effects in adolescent alcohol use is the failure to account for the co-evolution of network ties and alcohol use behavior. Assuming network ties are fixed while estimating changes in drinking produces biased parameter estimates. In a similar manner, modeling alcohol use as constant when estimating friendship tie formation can lead to systematic error in results. In addition, previous studies are limited in their control for social network elements beyond the unidirectional dyadic relationship which could drive friendship formation and behavior change. These factors include reciprocity (i.e., the likelihood to reply to friendship with friendship) and transitive closure (i.e., the likelihood of friends of friends to become friends). Furthermore, previous modeling strategies fall short in accounting for the dependent nature of social network ties data. Peer interactions that encompass interdependent selection and influence effects violate the notion of independence required by traditional modeling techniques such as structural equation or fixed effects modeling. The complexity of longitudinal social network data necessitates more advanced statistical methods than were used in previous studies of adolescent alcohol use.

A new analytical approach to the analysis of the co-evolution of social network ties and behavior is stochastic actor-based modeling [21-23], which provides a powerful new tool to simultaneously model an agent’s selection of friends based on alcohol use and changes in an agent’s alcohol use behavior over time. The primary assumptions of the actor-based model are that individuals choose their friendship ties and their behaviors in one step-at-a-time micro-steps. At each micro-step, an agent maximizes a personal utility function for surrounding network and relative friend behavior. At that time point, the agent only considers the current network characteristics in deciding whether a change in behavior or network tie is preferable to the current state. In this manner, the process of co-evolution of network and behaviors from one wave of data to another is simulated as a result of a potentially large number of individually unobserved micro-step changes, and the network and behavior preferences parameters can be estimated. The actor-based model can disentangle selection and influence and determine their relative contribution to similarities in alcohol use behavior among friends [23-25].

Recent studies have begun to use actor-based modeling to examine the relative contribution of selection and influence effects on adolescent alcohol use [26-30]. In one such study, a sample of 1,204 7^th^ graders in Finland were followed for 30 months to determine the degree to which the children selected or were influenced by friends based on alcohol use. The results indicated both selection and influence played a role in alcohol use similarities among friends, although influence was stronger at younger ages and selection became stronger as students aged [30]. Another study followed cohorts of 4th graders, 7th graders, and 10th graders in Sweden for 2 years. The findings indicated that selection based on alcohol use was strongest in early adolescence, while both influence and selection effects contributed to alcohol use similarities during later adolescence [29]. More research is needed to determine the relative role of selection and influence in alcohol use homogeneity within adolescent friendships. The current study will add to this body of literature by examining alcohol use behavior in a large sample of 7th through 11th grade U.S. adolescents.

Without clear guidance on the causal pathways between peers and alcohol use, adolescent alcohol interventions may be incomplete [9,31,32]. To fill this gap in the literature, the present study will investigate the selection and influence processes as they relate to peer friendship formation and alcohol use behavior in the larger context of adolescent friendship networks. Specifically, the study will address the following research questions:

Selection Research Question #1 Do adolescents select friends with similar alcohol use?

Influence Research Question #2 Do adolescents adjust their alcohol consumption in correspondence with the alcohol consumption level of their friends?

We hypothesize that both selection and influence effects will be present in the network model of adolescent alcohol use.

## Methods

### Data source

This study analyzes data from the National Longitudinal Study of Adolescent Health (Add Health). The Add Health study used stratified sampling to choose high schools and middle schools which were representative of US schools nationwide based on region of the country, urbanicity, school funding, and racial composition [33].

To construct the Add Health study sample, initially, all 7th through 12th graders at the 132 participating schools were invited to complete an In-School Survey. Students completing the In-School Survey (n = 90,118) were then eligible for the longitudinal portion of the study, which began with an in-home interview and parent survey. A random sample of 20,745 students selected from the In-School Survey respondents completed a Wave 1 in-home survey, which was administered between April and December, 1995. The Wave 1 survey collected data on social and demographic characteristics of the respondents, education and occupation of parents, household structure, risk behaviors including alcohol use, expectations for the future, self-esteem, health status, school-year extracurricular activities, and friendships. As part of the in-home survey, students selected their five best male and five best female friends from a complete school roster.

Approximately one year later, Wave 1 participants who had not yet graduated from high school were recontacted for a Wave 2 in-home survey. The Wave 2 in-home survey (n = 14,738) took place between April and December, 1996. The content of the Wave 2 survey was similar to data collected in Wave 1, including frequency of alcohol consumption over the past year. As part of Wave 2, students were again provided with a complete school roster and asked to name their five best male and five best female friends. The Wave 2 cohort excluded all subjects who were in their final year of high school at Wave 1.

The Add Health sampling design included saturated sampling of students at 14 schools. All students in attendance at these schools at the time of the Wave 1 survey were included in the sampling frame for the in-home survey. The inclusion of all students from a school allows the investigation of complete peer network structures and their influence on behaviors and life choices.

### Analysis sample

The study sample for the present analysis includes 2,563 Add Health subjects from the saturated school subsample. Subjects who completed the Wave 1 in-home interview but were non-responders at Wave 2 were included in the model using the SIENA imputation approach [21]. In this approach, outgoing ties of non-responders are imputed and treated as non-informative for statistical calculations using last observation carry forward while incoming ties are allowed to vary and to contribute to the estimation procedures. The sample excluded subjects who did not name or were not named by at least one friend at either Wave 1 or Wave 2.

Low network stability between study waves could throw doubt on the reliability of the friendship data reported and may increase the likelihood of convergence failure in the iterative estimation process. The Jaccard index is a measure of network stability between study waves [34], indicating the proportion of existing friendship ties within each school which remain consistent from Wave 1 to Wave 2 out of the total number of ties reported at either wave. The index is calculated as:

(1) $$J=\left(F_{11}\right)/\left(F_{11}+F_{01}+F_{10}\right)\text{,}$$

where F_11_ is the number of ties present at both waves, F_01_ is the number of new ties formed in Wave 2, and F_10_ is the number of ties dissolved between Wave 1 and Wave 2. A Jaccard Index of 0.20 or higher for the school is generally required for inclusion in a stochastic actor-based model analysis [21]. One school from the saturated subsample was excluded from the analysis based on a Jaccard index of less than 0.20. A possible explanation for low network stability is that friendships change when students transition from a middle school to high school setting. A total of 13 schools were included in the analysis.

### Measures

Variables were chosen a priori based on previous findings in the adolescent alcohol literature [11-20,35].

### Alcohol use

On the in-home survey, students answered the question: “How often did you consume alcohol in the past year?” Categorical responses included never, 1 or 2 times, 3 to 12 times, monthly but not weekly, weekly, and more than once a week.

### Social networks

At both Wave 1 and Wave 2 of the study, students provided responses to: “Name your 5 best male and 5 best female friends from your school roster.” Social networks within each school resulted from the formation of a friendship matrix based on the directed friendship designations.

### Demographics

Students provided age, grade, and race/ethnicity data at the in-home interview. Age was calculated to the nearest month.

### Family characteristics

Our previous studies of the Add Health data show that family bonding is a strong predictor of adolescent alcohol use [36]. Study participants answered: “On a scale of 1 to 5, how often do you and your family have fun together?” This item on family fun represents the notion of family bonding in Add Health. In addition, parents of the sample students indicated in a parent interview conducted at Wave 1 how often they drank alcohol in the past year.

### Statistical analysis

#### Stochastic actor-based modeling

The analysis uses stochastic actor-based modeling to assess the co-evolution of alcohol use and friendship ties from Wave 1 to Wave 2 of the study [21]. The stochastic actor-based model assumes changes in the network take place according to a continuous-time Markov chain with stationary transition distribution. Changes from Wave 1 to Wave 2 occur through mini-steps where the future state of the network is dependent only on the present state. Each mini-step is evaluated by choosing a random student *i* among all network members and either a potential friendship change or behavior change. In a potential friendship change, the student *i* might change an outgoing tie to student *j* so as to maximize the objective function for network structure and a random unexplained influence:

(2) $$f_{i}^{X}\left(\beta ,x\left(i\mapsto j\right),z\right)+U_{i}^{X}\left(t,x,j\right)$$

where β is the parameter set, *x(i → j)* is the network changes that would occur if the tie between individual *i* and individual *j* in the network were changed, *z* is the state of behaviors within the network, and *U* is an independent random component. For a given actor *i* (the actor who takes the micro-step), the objective function is maximized over all potential alters *j*. The β parameters are estimated by Method of Moments (MoM). The MoM algorithm compares the observed network (obtained from the data) to hypothetical networks generated through repeated Monte Carlo simulations [22].

Similarly, the individual *i* might make a one-step change in alcohol-use behavior based on the objective function for the parameterized alcohol outcome and a random influence:

(3) $$f_{i}^{Z}\left(\beta ,x,z\left(i\right)\right)+U_{i}^{Z}\left(t,z,i\right)$$

where β is the parameter set for behavior changes, *x* is the current state of network ties, *z(i)* is the next potential behavior state that would occur after a micro-step change, and *U* is an independent random component. By estimating the β parameters in the model, the simulated analysis seeks to define what tendencies and trends influence changes in friendship ties and in alcohol-use behavior. The study model simultaneously evaluates the evolution of the adolescent friendship network and adolescent alcohol drinking while controlling for age, gender, race/ethnicity, parental drinking, and family bonding. The study uses the statistical program RSIENA (Simulated Investigation for Empirical Network Analysis) [21], originally designed by Snijders and van Duijn [37] and programmed by Ruth Ripley and Krists Boitmanis.

### Model specification

The model specification consists of two parts, friendship network evolution and alcohol use behavior evolution. First, the *friendship network evolution* portion of the model identifies the preferred choices in friendship ties depending on a list of friendship choice variables. Friendship choice variables are based on current network structure and friend attributes at each iterative step of the simulation process. Three alcohol-related variables are included in the friendship evolution part of the model: (1) the effect of an adolescent’s alcohol use behavior on number of friends chosen (alcohol use ego), (2) the effect of an adolescent’s alcohol drinking on the probability of being chosen as a friend by others (friend alcohol use), and (3) the effect of similar alcohol consumption on friendship selection (alcohol use similarity).

Several characteristics of the friendship network structure are included as control variables [21,38,39]. These take account of the overall density of friendship ties within the network (density), the likelihood to reciprocate friendship nominations (reciprocity), the tendency for friends of friends to be friends (transitive triplets), the propensity for closure in three-person friendships (3-cycles), the propensity for individuals with more in-coming friendship nominations to attract further friendship nominations (in-degree popularity), the tendency for individuals who name many others as friends to attract friendship nominations (out-degree popularity), and the inclination for students who name more friends to generate even more out-going friendship ties over time (out-degree activity). Control variables are age, gender, and race/ethnicity effects on the number of friends chosen, on the probability of being chosen as a friend, and on the likelihood of friends being of similar age, gender, and race/ethnicity.

Second, the *alcohol use behavior evolution* portion of the model specifies a list of variables that could influence potential alcohol drinking behavior changes. The model contains one main friendship-related influence component: the tendency for alcohol use to change based on the average drinking of immediate friends. Control variables include age, gender, race/ethnicity, parental drinking, family bonding and linear and quadratic shape effects modeling average drinking across the network.

We employ both the Snijders-Baerveldt meta-analysis (two-sided) test [40] and the Fisher’s combination procedure [41] to test overall significance of the primary and control variables across schools. The Snijders-Baerveldt test makes inference about parameters in the population of schools from which the studied schools are considered to be a sample, while the Fisher’s combination procedure makes inference only about the particular schools in the study. The Fisher’s procedure provides two tests, a right-sided test that the overall variable effect is positive and a left-sided test that examines if the overall variable effect is negative. To control for the multiple testing (right and left), we consider an effect to be significant if either of the combination tests was significant at level p ≤ .025. In the case of conflicting Snijders-Baerveldt and Fisher’s combination test results, we lean toward accepting the Fisher’s test due to the variation in school sizes in the sample.

## Results

The sample consisted of 2,563 adolescents in grades 7 through 11 at Wave 1 of the Add Health survey (Table 1). The participants included 1,301 (51%) boys and 1262 (49%) girls. Mean age was 15.8 years. Two fifths (39%) of respondents were minorities, with 16% African American, 18% Hispanic, and 3% Asian. Forty-four percent of the adolescents’ parents reported drinking alcohol in the past year. Over 70% of the adolescents reported strong family bonding by indicating that they had fun with their family quite a bit or very much. The Wave 2 sample included 2,299 adolescents, 89.6 percent of the original sample. Subjects lost to follow-up between Wave 1 and Wave 2 were more likely than Wave 2 respondents to be weekly alcohol users at Wave 1 (13% vs 9%, p = 0.040). There were no significant differences between respondents and non-respondents in age, grade, gender, race/ethnicity, or family bonding.

**Table 1:** Descriptive Statistics of Add Health Sample, Wave I, 1995 (n = 2,563)

***Characteristic***	
*Demographics*	
Male (%)	50.8
Age, mean (sd) (years)	15.8 (1.3)
Age, range (years)	12-18
Grade Level (%)	
7th grade	7.0
8th grade	7.3
9th grade	17.5
10th grade	34.7
11th grade	33.4
Race (%)	
Non-Hispanic white	60.9
Black	16.4
Native American	1.6
Asian	3.4
White Hispanic	17.7
*Family Characteristics*	
Parent alcohol consumption, past 12 months (%)	
None	56.2
1-2 times	27.1
3-12 times	7.4
More than monthly, less than weekly	5.6
Weekly or more often	3.7
Family has fun together (quite a bit/very much, %)	60.0

Characteristics of the 13 schools in the study and alcohol use within the schools are reported in Table 2. The mean number of students per school was 197. At Wave 1, the average number of friendship nominations to other students in the same school was just over two per student. Fifty percent of students reported no alcohol use in the past 12 months at Wave 1. Alcohol abstainers increased to 54 percent at Wave 2. The proportion of monthly or weekly drinkers increased between waves, from 18 percent at Wave 1 to 19.6 percent at Wave 2. Just over half of the students reported the same drinking frequency at both waves of the study. Drinking changes from Wave 1 to Wave 2 were equally balanced with 24 percent of respondents reporting an increase in drinking frequency and 24 percent reporting a decrease.

**Table 2:** Network Statistics for the Add Health Schools (n = 13) in the Analysis Sample

	***Wave 1(n = 2563)***	***Wave 2 (n = 2299)***
***Network Structure***		
Mean number of adolescents per school (range)	197.2 (48-987)	176.8 (41-889)
Mean number of friendship nominations per student	2.04	1.85
Reciprocal friendships (%)	22.9	25.3
Jaccard Index^a^, mean (range)	.253 (.200-.325)	
***Individual Characteristics***		
Alcohol use, past 12 months		
None (%)	49.6	54.0
1-2 times (%)	18.3	14.1
3-12 times (%)	14.2	12.3
More than monthly, less than weekly (%)	8.3	8.7
Weekly or more often (%)	9.6	10.9

^a^Jaccard network stability index = (Number of ties present at both Wave 1 and Wave 2) / (Number of ties present at both Wave 1 and Wave 2 + Number of new ties + Number of dissolved ties).

Table 3 presents the *friendship network evolution* from Wave 1 to Wave 2. Friendship selection was associated with similarity in alcohol consumption (*p* < .001), while significant control mechanisms included reciprocity (*p* < .001), transitive triplets (*p* < .001), 3-cycles (*p* = .012), in-degree popularity (*p* < .001) and out-degree popularity (*p* < .001). Students were more likely to choose as friends other students of similar age (*p* = .002), gender (*p* < .001), and race/ethnicity (*p* < .001). The number of out-going friendship nominations was not associated with more frequent alcohol consumption (adolescent alcohol use), but greater number of in-coming friendship nominations (friend alcohol use) was correlated with increased alcohol use (*p* = .006).

**Table 3:** Stochastic Actor-Based Model Results for Network Selection

	**β**	**SE(β)**	***p*-value**^**a**^	**Between school std. dev.**	**Fisher’s combination 1-side test**	
					**Right-side (+)**	**Left-side (-)**
**Density**	**-3.42**	**0.38**	**<.001**	1.91	1.000	**<.001**
**Reciprocity**	**2.53**	**0.20**	**<.001**	0.95	**<.001**	1.000
**Transitive triplets**	**0.84**	**0.09**	**<.001**	0.31	**<.001**	1.000
**3-cycles**	**-0.44**	**0.14**	**.012**	0.56	.997	**<.001**
**In-degree popularity**	**0.09**	**0.01**	**<.001**	0.08	**<.001**	1.000
**Out-degree popularity**	**-0.16**	**0.03**	**<.001**	0.18	1.000	**<.001**
Out-degree activity	-0.04	0.03	.197	0.16	.658	**<.001**
Adolescent age	-0.10	0.05	.097	0.31	.895	**<.001**
Friend age	0.05	0.03	.120	0.15	**.012**	.796
**Age similarity**	**1.48**	**0.40**	**.002**	1.81	**<.001**	1.000
Adolescent male	0.04	0.09	.664	0.84	.031	.423
**Friend male**	**0.11**	**0.04**	**.016**	0.45	**.005**	.995
**Gender same**	**0.36**	**0.05**	**<.001**	0.33	**<.001**	1.000
Adolescent minority	0.06	0.07	.408	0.30	.585	.725
Friend minority	-0.14	0.25	.608	0.99	.733	.145
**Same race**	0.58	0.43	.24.1	1.33	**<.001**	.991
Adolescent alcohol use	0.03	0.03	.361	0.25	.212	.767
**Friend alcohol use**	**0.08**	**0.02**	**.001**	0.18	.**006**	1.000
**Alcohol use similarity**	**1.28**	**0.21**	**<.001**	0.82	**<.001**	1.000

^a^Snijders-Baerveldt two-sided test [39].

Parameter estimates β and standard error for stochastic actor-based model of the evolution of school friendships in the Add Health study. Coefficients correspond to the change in log-odds of a friendship nomination being present. Characteristics are bolded when the Fisher’s combination test yields a p-value less than 0.025.

The significant beta coefficient for alcohol use similarity in the network selection part of the model (β = 1.28) is comparable to a log-odds ratio of friendship formation in a logistic regression analysis theoretical framework. Exponentiation of the beta coefficient produces an odds-ratio of 3.60, indicating that a friendship nomination between two students who share the same alcohol use frequency is 3.60 (95% CI: 2.01-9.62) times more likely to occur than an otherwise identical friendship between two students who are maximally different with respect to alcohol use. Adolescents are more likely to nominate as friends others who drink similarly to themselves, which is consistent with a selection effect.

**Figure 1:**
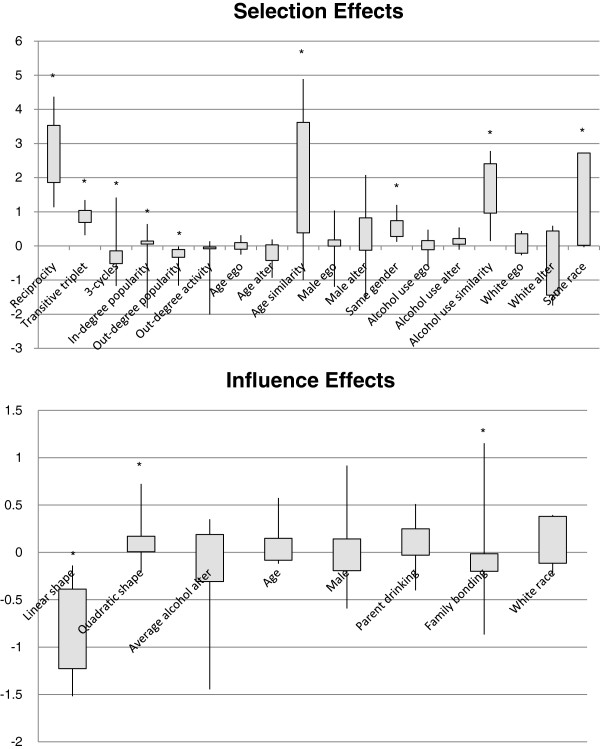
**Boxplots of Selection and Influence Effects across Schools.** Boxplots (Minimum, Maximum, 25th and 75th percentiles) for selection and influence effect parameter estimates β across all schools (n = 13). Significant coefficients are labeled with an asterisk, where a co-efficient is considered significant if the Snijders-Baerveldt test is less than 0.05 or the Fisher combination test is less than 0.025. For selection effects, coefficients correspond to the log-odds of a friendship tie being present vs. absent if the selection criterion is met. For influence effects, coefficients correspond to log-odds of a one-step increase in alcohol use frequency given a one-unit increase in the independent variable.

Figure 1 displays the range, 25th percentile, and 75th percentile for β parameter estimates of friendship network evolution parameters across the 13 schools in the study over 1-year follow-up. The dominant features of friendship evolution are reciprocity, transitivity, and age, race, and alcohol use similarity.

Table 4 presents the results of the *alcohol behavior evolution* portion of the model from Wave 1 to Wave 2. Interestingly, more frequent drinking by immediate friends was not significantly associated with increased frequency of alcohol consumption (*p* = .139). The stochastic actor-based model results do not support a significant influence effect based on alcohol consumption. In terms of the other covariates included in the model, family bonding was a significant protective factor for alcohol use (β = -0.06, *p* = .009). Frequency of parental alcohol use was not significantly associated with alcohol consumption frequency by the adolescent (*p* = .220).

**Table 4:** Stochastic Actor-Based Model Results for Influence Effects on Alcohol Use Behavior

	**β**	**SE(β)**	***p*-value**^**a**^	**Between school std. dev.**	**Fisher’s combination 1-side test**	
					**Right-side(+)**	**Left-side(-)**
**Linear shape parameter**	**-0.60**	**0.09**	**<.001**	0.34	1.000	**<.001**
**Quadratic shape parameter**	**0.07**	**0.02**	**.003**	0.08	**<.001**	.999
Average friend alcohol use effect	0.07	0.04	.139	0.18	.498	.780
Age effect	0.02	0.03	.456	0.13	.059	.733
Gender effect (male)	0.07	0.06	.252	0.24	.101	.667
Race effect (non-white)	0.04	0.05	.489	0.14	.330	.914
Parental drinking effect	0.03	0.03	.220	0.10	.103	.882
**Family bonding effect**	**-0.06**	**0.02**	**.009**	0.08	.999	**.011**

^a^Snijders-Baerveldt two-sided test [39].

Parameter estimates β and standard error for stochastic actor-based model of change in alcohol use in the Add Health study. Alcohol use frequency measured on a 0 (never) to 4 (weekly or more often) scale. Coefficients correspond to the change in log-odds of increased drinking frequency given a one-unit increase in the independent variable. Characteristics are bolded when the Fisher’s combination test yields a p-value less than 0.025.

## Discussion

The main objective of this investigation is to disentangle the selection and influence processes governing peer relationship’s impact on adolescent alcohol use. To achieve this goal, we utilize a stochastic actor-based model to analyze the dynamic interplay of friendship formation and alcohol behavior changes as they co-evolve over time. Specifically, the study evaluates if similar alcohol use among friends is more likely a result of a tendency for adolescents to choose friends with similar alcohol use behavior, or as a result of teen influence on each other’s drinking.

The results demonstrate that selection effects in adolescent friendships are based, in part, around commonalities in alcohol use behavior. Homophily in friendship formation is also based on age, gender, and race/ethnicity. The analysis controls for reciprocity (i.e. tendency to have reciprocal friendships), transitivity (i.e. tendency to become a friend of a friends’ friend), and degree effects (i.e., number of in-coming or out-going friendship nominations) as drivers of friendship formation. Our results are in line with previous studies showing selection effects to be a strong factor in alcohol use similarity within adolescent friendships [27-30]. Our study is the first to employ agent-based modeling for disentangling peer selection and influence effects on alcohol use behaviors in a sample of U.S. middle and high school students.

The study findings offer little support for influence effects among teens after the social ties with their peers are in place. Our findings suggest that friends share similar alcohol behaviors not because they adjust to the behavior of one another, but because they selected each other as friends to some degree because of similar alcohol use patterns, or of similarity in their behaviors associated with alcohol use. The results are in contrast to two European studies that identified both selection and influence as significant in alcohol use similarities among adolescent friends [29,30]. Our study population was predominantly 10^th^ and 11^th^ graders, which may have contributed to the differing outcomes. Prior studies noted that friendship selection played a relatively stronger role than peer influence when explaining similarity of early adolescent friends’ alcohol use and that influence effects tended to diminish as students aged [27,30].

Interestingly, we found evidence of close family bonds, defined here as having family fun, to be negatively associated with alcohol intake. These data demonstrate that strong family ties may offer protective benefits against adolescent alcohol consumption.

Our results argue that homophily limits an adolescent’s social interactions in a way that has powerful implications for the information they receive and the attitudes they form toward alcohol use. Homophily creates strong divides in adolescent social environments, which set the stage for reinforcing alcohol use norms. More research is needed to evaluate the dynamics of social network and alcohol use change over time. Future studies may need to explore how modifications of social networks could affect adolescent alcohol abuse.

The strength of the study lies in its innovative methodology, wealth of friendship variables, prospective design and large study sample size. The study also has several limitations. First, adolescents were limited to nominate only up to 10 friends which may have obscured the friendship formation parameters in the model. However, other studies show that students on average report having four friends [27,30]. Second, the analysis, by limiting itself to friendships within a school, may not have captured all peers in the adolescent social network. The average number of friends reported in the sample was approximately two, fewer than in other studies [27,30]. This implies that potentially only some of the influential peers were nominated, which limits the conclusions that can be drawn. However, school-based networks may be most pertinent for intervention efforts. Third, alcohol use in the study was self-reported. However, self-reported alcohol use is generally considered to be a valid measure among adolescents. Add Health used a computer assisted data entry process for sensitive questions such as alcohol and drug use to protect confidentiality and enhance full reporting. Fourth, we only examined alcohol, and not tobacco and other drug use, in the analyses. It may be the case that peer selection occurred not specifically for alcohol, but for other substance use as well. It is also possible that other peer characteristics (e.g., peer delinquency) may precede adolescent alcohol use and be related to both peer selection and influence. Fifth, loss to follow up, in particular among the heavier drinkers from Wave 1, may have influenced the results. The increase in alcohol abstainers over the course of one year, which is in contrast to longitudinal studies showing higher prevalence of drinking with increasing age [42], suggests there may be limits to the generalizability of the results. The 44% frequency of parental alcohol consumption also raises questions about the representativeness of the sample, as national surveys indicate that 60%-70% of adults are alcohol drinkers [42]. Parents of adolescents in the study may have underreported their current drinking. Sixth, the selection and influence processes may depend on contextual and cultural aspects of the schools analyzed, which limits generalizability to different school contexts and cultural settings. Finally, the study does not directly address whether the peer selection and influence processes operate in a similar manner for alcohol initiation or for heavy drinking. Future studies could investigate the role of peer interactions in alcohol initiation and heavy drinking.

## Conclusions

This investigation demonstrates that network selection, or homophily, plays a prominent role in adolescent alcohol drinking similarities among friends. Understanding the dynamics through which adolescent friendships and alcohol use are initiated and maintained will allow for designing adolescent alcohol abuse intervention strategies to groups or subgroups of students. Our findings may be of interest to parents, health care professionals, school administrators, law enforcement and community leaders who focus on alcohol prevention efforts.

## Competing interests

The author(s) declare that they have no competing interests.

## Authors’ contributions

MM conceived of the study, participated in the design, ran the analyses, and helped draft the manuscript. LM participated in the study design, and helped draft the manuscript. LZ participated in the study design, and helped draft the manuscript. All authors read and approved the final manuscript.

## Pre-publication history

The pre-publication history for this paper can be accessed here:

http://www.biomedcentral.com/1471-2431/12/115/prepub

## Author details

^1^Department of Family Medicine, University of Wisconsin School of Medicine and Public Health, 1100 Delaplaine Ct, Madison, WI 53715, USA. ^2^Department of Primary Care Health Promotion, Maastricht University (Netherlands) School for Public Health, PO Box 616, 6200, MD Maastricht, the Netherlands.
